# Editorial: Advanced Theranostic Nanomedicine in Oncology

**DOI:** 10.3389/fbioe.2020.00142

**Published:** 2020-02-25

**Authors:** Christos Tapeinos, Attilio Marino, Gianni Ciofani

**Affiliations:** Smart Bio-Interfaces, Istituto Italiano di Tecnologia, Pisa, Italy

**Keywords:** nanoparticles, nanofibers, oncology, smart theranostics, glioblastoma

The Research Topic entitled “Advanced Theranostic Nanomedicine in Oncology” presents a series of articles that describe the latest research updates of biologically-inspired nanostructures used in the field of oncology. In addition, it briefly describes the biological mechanisms of certain cancers and how we can take advantage of these mechanisms to improve the therapeutic efficacy of future nano-therapeutics. With this issue, we aim to raise awareness for the challenges in the common ground between the fields of oncology and biomedical engineering, and also to present up-to-date counter-challenging research achievements. The issue is comprised of 12 selected peer-reviewed manuscripts (8 reviews and 4 original research articles) derived from the fields of (bio)materials science and engineering, chemistry, physics, and biology.

A variety of smart nanomaterials, including carbon-based nanofibers (Sayin et al.), multifunctional liposomes (Formicola et al.), polymeric nano-constructs (Ferreira et al.), and inorganic nanoparticles (Hossain et al.) are presented through original research works. These novel and smart nanomaterials with tailor-made characteristics have been carefully designed, aiming at improving their therapeutic efficacy while in tandem to reduce the side-effects of the chemotherapeutics that they encapsulate.

The first example of these smart nanomaterials is given by polyvinyl alcohol (PVA)-based nanofibers made through electrospinning by Sayin et al.. In this work, the fabricated nanofibers presented excellent biocompatibility and a pH-controlled release of the encapsulated Rose Bengal drug model. The nanofibers also demonstrated an increase in reactive oxygen species (ROS) production that led to the apoptosis of U87 glioblastoma cells.

A different approach to the treatment of glioblastoma is also presented by Formicola et al.. In this study, multifunctional liposomes made of cholesterol/sphingomyelin and surface functionalized with mApoE and chlorotoxin peptides (Mf-LIP) were used as a model nano-vehicle to study the formation of tunneling nanotubes, and the effect of these nanotubes in the delivery of chemotherapeutics. The comparison of the U87 MG cell line with a standard human astrocytic cell line demonstrated that he U87 MG cells form almost exclusively thick and long protrusions, whereas healthy astrocytes formed thinner and shorter tunneling nanotubes. This suggested that nanotubes are potentially useful as drug-delivery channels for cancer therapy especially for isolated tumor niches that cannot be targeted through simple chemotherapeutic diffusion.

Discoidal polymeric nano-constructs consist of a class of nano-theranostic agents with tunable characteristics including high biocompatibility, controllable size, loading capacity, and stimuli-responsive release. In the presented work of Ferreira et al. food and drug administration (FDA)-approved poly (lactic-*co*-glycolic acid) was used for the fabrication of discoidal nano-constructs, which were subsequently optimized to increase their loading efficiencies. Two different loading methodologies (direct vs. absorption) and compound attributes (hydrophobicity and molecular weight) have been studied. *In vitro* (breast cancer cells) and preliminary *in vivo* studies demonstrated that the properties of these nano-constructs could be finely tuned and improve their overall therapeutic performance.

The final original research presented in this topic by Hossain et al. is related to the investigation of the antibacterial activity of biogenic silver nanoparticles. Despite the fact that this research is not directly related to the field of oncology, it presents valuable data on the fabrication, characterization, and antibacterial activity of biocompatible silver nanoparticles that have already been reported elsewhere as potential anticancer agents.

Besides the original research manuscripts, this topic presents a series of review papers that summarize the recent advances in the fields of nanomedicine and oncology. As a starting example, the application of high-intensity ultrasound as a non-invasive tumor-ablation method, its immunomodulatory action, and its effects in drug delivery are discussed by Tharkar et al.. In continuation, theranostic applications based on gold nanoparticles and their effect depending on the heterogeneity of tumors is discussed by Roma-Rodrigues et al., while a variety of general nano-theranostics for the treatment of pancreatic adenocarcinoma are described by Brachi et al..

The increasing development and the numerous applications of two-dimensional (2D) nanostructures in various fields, including cancer therapy and diagnosis, and more specifically in the field of photodynamic therapy, is presented by Gazzi et al.. Carrying on the field of 2D nanomaterials, the synthesis, the surface modification strategies, the biocompatibility, and the bioapplications of 2D boron nitride nanostructures are reported by Emanet et al.. Theranostic nanomedicine approaches for the treatment of glioblastoma are detailed by d'Angelo et al., while nanomaterials-based combinational chemo-photothermal therapy is introduced by Li et al..

Finally, Natoni et al. elucidate the mechanisms behind sialylation and multiple myeloma, and how the inhibition of sialylation may represent an advanced therapeutic strategy able to overcome the bone marrow-mediated chemotherapy resistance. In addition, different approaches that allow the delivery of sialylation inhibitors to the bone marrow microenvironment are also analyzed.

Summarizing, we hope that this collection of the-state-of-the-art articles and reviews will provide insights into the limitations on the field of nanomedicine, and will promote ideas on how to overcome these limitations for successfully developing enhanced therapeutic strategies. Additionally, we anticipate that this Research Topic will constitute a convenient and beneficial guide toward early-stage and senior researchers in the field of biomedical engineering.

## Author Contributions

CT wrote the editorial, which was revised, proofed, and accepted by all the authors.

### Conflict of Interest

The authors declare that the research was conducted in the absence of any commercial or financial relationships that could be construed as a potential conflict of interest.

